# Multi-site enhancement of osteogenesis: peptide-functionalized GelMA hydrogels with three-dimensional cultures of human dental pulp stem cells

**DOI:** 10.1093/rb/rbae090

**Published:** 2024-08-10

**Authors:** Leyi Liang, Shuze Wang, Xiyue Zhang, Tao Yan, Xiyun Pan, Yuzhong Gao, Xing Zhang, Qiang Wang, Liu Qu

**Affiliations:** Liaoning Provincial Key Laboratory of Oral Diseases, School and Hospital of Stomatology, China Medical University, Shenyang, Liaoning 110001, China; Liaoning Provincial Key Laboratory of Oral Diseases, School and Hospital of Stomatology, China Medical University, Shenyang, Liaoning 110001, China; Liaoning Provincial Key Laboratory of Oral Diseases, School and Hospital of Stomatology, China Medical University, Shenyang, Liaoning 110001, China; Department of Orthopedics and Sports Medicine and Joint Surgery, The People's Hospital of China Medical University, Shenyang, Liaoning 110016, China; Liaoning Provincial Key Laboratory of Oral Diseases, School and Hospital of Stomatology, China Medical University, Shenyang, Liaoning 110001, China; Department of Orthopedics, The First Affiliated Hospital of Jinzhou Medical University, Jinzhou, Liaoning 121001, China; School of Materials Science and Engineering, University of Science and Technology of China, Hefei, Anhui 230026, China; Liaoning Provincial Key Laboratory of Oral Diseases, School and Hospital of Stomatology, China Medical University, Shenyang, Liaoning 110001, China; Liaoning Provincial Key Laboratory of Oral Diseases, School and Hospital of Stomatology, China Medical University, Shenyang, Liaoning 110001, China

**Keywords:** gelatin methacryloyl hydrogel, bone-forming peptide-1, multi-site bone regeneration, human dental pulp stem cells

## Abstract

Human dental pulp stem cells (hDPSCs) have demonstrated greater proliferation and osteogenic differentiation potential in certain studies compared to other types of mesenchymal stem cells, making them a promising option for treating craniomaxillofacial bone defects. However, due to low extracting concentration and long amplifying cycles, their access is limited and utilization rates are low. To solve these issues, the principle of bone-forming peptide-1 (BFP1) *in situ* chemotaxis was utilized for the osteogenic differentiation of hDPSCs to achieve simultaneous and synergistic osteogenesis at multiple sites. BFP1-functionalized gelatin methacryloyl hydrogel provided a 3D culture microenvironment for stem cells. The experimental results showed that the 3D composite hydrogel scaffold constructed in this study increased the cell spread area by four times compared with the conventional GelMA scaffold. Furthermore, the problems of high stem cell dosage and low rate of utilization were alleviated by orchestrating the programmed proliferation and osteogenic differentiation of hDPSCs. *In vivo*, high-quality repair of critical bone defects was achieved using hDPSCs extracted from a single tooth, and multiple ‘bone island’-like structures were successfully observed that rapidly induced robust bone regeneration. In conclusion, this study suggests that this kind of convenient, low-cost, island-like osteogenesis strategy involving a low dose of hDPSCs has great potential for repairing craniomaxillofacial critical-sized bone defects.

## Graphical abstract

**Graphical Abstract:**
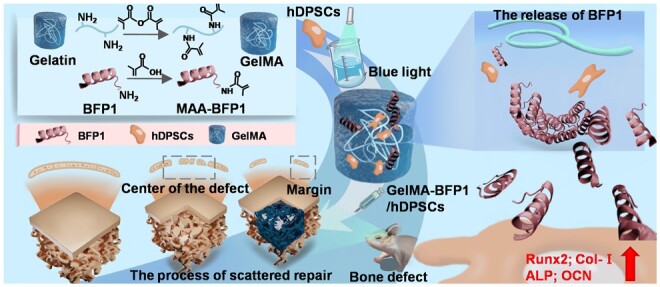

## Introduction

Stem cell therapy can effectively repair large craniomaxillofacial bone defects [1, 2]. Among these, human dental pulp stem cells (hDPSCs) are preferred seed cells because of their tissue homology and accessibility [3–5]. However, current hDPSCs-based therapies are hindered by various factors, including immune rejection at both low and high doses [6]. Therefore, further in-depth studies are needed to maximize the role of hDPSCs in promoting osteoinductive accelerated defect repair.

Conventional bone repair usually involves a peripheral-center model, which is characterized by the presence of stem cells on the surface of bone defects that gradually spread towards the center during bone regeneration [7]. However, this model often leads to inadequate bone-repair efficiency. Even with an increased rate of stem cell loading, achieving a proportional osteogenic effect remains challenging. Additionally, the cells within the scaffold may encounter challenges such as low survival rates due to excessive density, limited porosity within the scaffold and inefficient material exchange, these factors collectively contribute to suboptimal repair outcomes [8, 9].

The effect of bone formation was not proportional to the increase in the stem cell-loading rate [10]. Therefore, it was hypothesized that the inefficacy of hDPSCs at a high dose was related to the spatial limitations of their distribution. Changing the distribution pattern of stem cells can influence the form of bone regeneration from a unidirectional peripheral-central osteogenesis to a multidirectional superposition of multiple osteogenic centers, which may be the most effective way to increase hDPSCs utilization and improve their osteoinductive properties. This approach may represent a new strategy for improving the utilization rate of hDPSCs and their osteoinductive properties. Another important issue to consider is the difficulty in achieving satisfactory results using stem cells alone to treat bone defects [11]. Therefore, enhancing the osteoinductive properties of hDPSCs is imperative for improving their efficiency in bone repair.

Bone-forming peptide-1 (BFP1) plays a key role in regulating bone formation by accelerating bone growth [12]. BFP1 is derived from the immature region of bone morphogenetic protein 7 (BMP-7), contains 15 amino acids, and is superior to BMP-7 in terms of its stability and ability to promote bone formation [13]. However, owing to the rapid clearance of the locally defective region of BFP1 and its limited bioavailability, free administration of BFP1 does not usually achieve the desired therapeutic effect and cannot regulate the entire bone-repair cycle [14, 15]. Therefore, it is believed that an effective controlled-release system can increase the efficacy of BFP1 by mimicking the real stem cell state *in vivo* and achieving long-lasting controlled release of the peptide.

Considering the rapid clearance rate of BFP1, an urgent need exists for an optimized sustained-release drug vehicle for long-lasting efficacy. Gelatin methacryloyl (GelMA) hydrogel, owing to its resemblance to the natural extracellular matrix, as well as its considerable drug-loading efficiency, is an ideal vehicle [16]. Previous studies investigated the mechanism of BFP1-stem cell interactions and concluded that the relative distributions of peptide-stem cell and stem cell-stem cell interactions were key for effective endocytosis [17, 18]. However, traditional self-assembled peptides often affect the osteogenic-induction efficiency of stem cells because of their uneven distribution. In addition, the porous structure and short diffusion distance of GelMA results in the rapid release of the encapsulated drugs, which poses challenges for their complete release during the early stage of osteogenesis throughout the bone-regeneration process [19, 20]. To circumvent these problems, GelMA was covalently modified with BFP1 to achieve a uniform distribution and controlled release, meeting the expected requirements for peptide delivery and stem cell bone defect therapy in a clinical setting.

**Figure 1.**
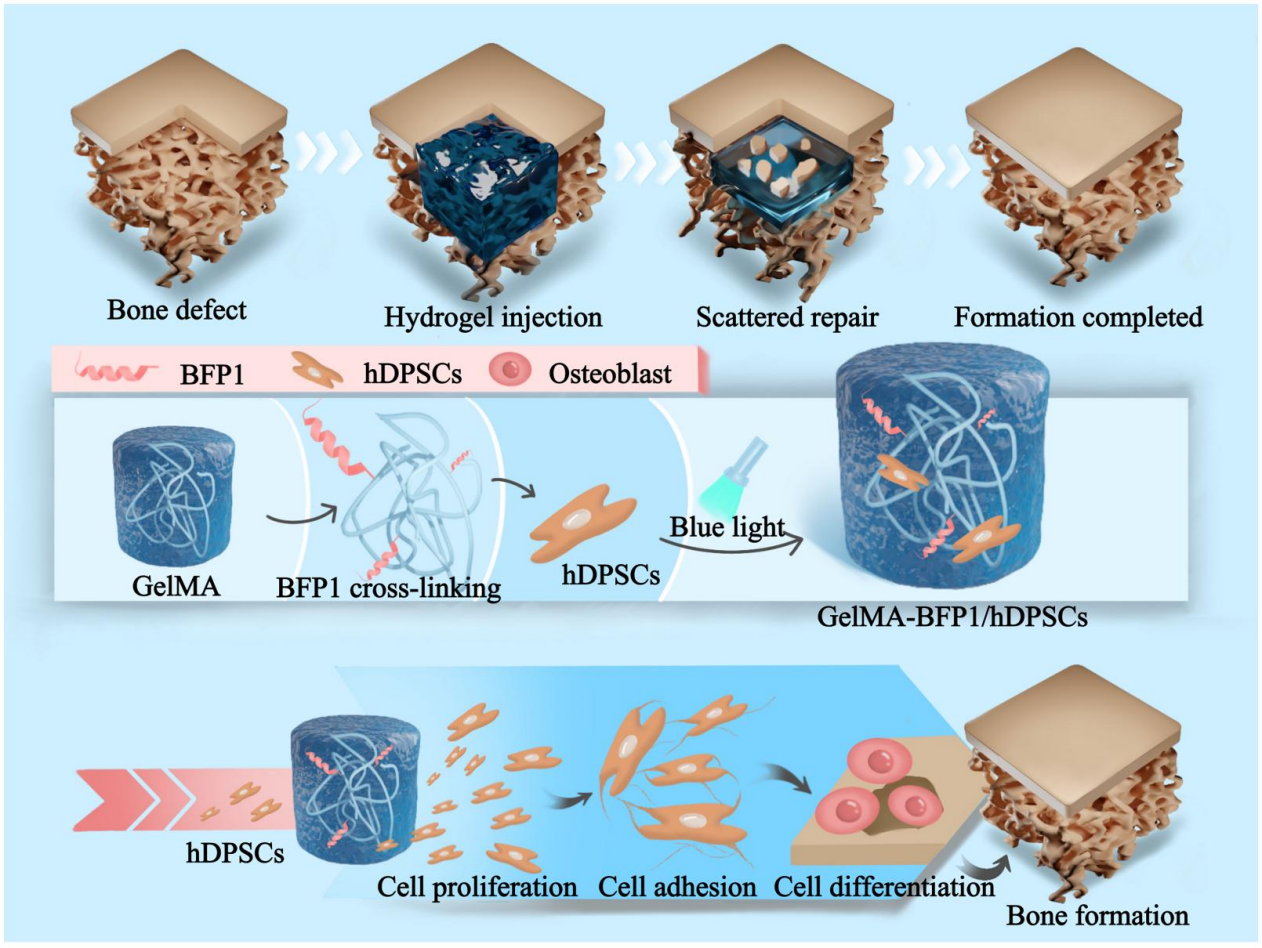
Schematic illustration of the process used to prepare a cell-laden GelMA-BFP1 hydrogel scaffold specifically designed to facilitate enhanced bone regeneration. The GelMA-BFP1 scaffold comprised GelMA hydrogel and MAA-modified BFP1 (MAA-BFP1), encapsulating hDPSCs to facilitate bone regeneration.

In this study, to address the underutilization of hDPSCs and the short-range release of BFP1, an injectable photocrosslinked hydrogel system (GelMA-BFP1/hDPSCs system) encapsulating hDPSCs for bone regeneration was developed using a GelMA hydrogel and methacrylic acid (MAA) modified BFP1 (Figure 1). A series of experiments were conducted to investigate the biological behaviors of hDPSCs with controlled BFP1 release in a 3D culture environment, including adhesion, proliferation and differentiation ability. Then, a series of *in vivo* experiments were performed to investigate the successful construction of a multi-site osteogenic strategy. We expect that this stem cell therapy will be a promising strategy to achieve higher stem cell utilization, greater range and more rapid and robust osteogenic effect in craniomaxillofacial bone repair.

## Materials and methods

### Sample preparation

#### Synthesis of gelatin methacrylate hydrogel

GelMA was synthesized as described [21]. Initially, 10 g of type B gelatin was added to 100 ml of phosphate-buffered saline (PBS) at a temperature of 50°C. The concoction was swiftly stirred for 1 h. Subsequently, 8 ml methyl acrylamide was gradually added to the gelatin solution with constant stirring at 50°C for 2 h. About 100 ml of preheated PBS was added to halt the reaction. The resulting mixture was placed in dialysis bags (12–14 kDa) and dialyzed against deionized water at 40°C for 1 week. The dialyzed GelMA was diluted with 200 ml warmed ultrapure water. Finally, the GelMA solution was transferred to 50 ml tubes and lyophilized for 7 days.

#### Preparing hydrogels load with bone-forming peptide-1 and methylacrylamide bone-forming peptide-1

About 50 mg lyophilized GelMA and 50 µg MAA-BFP1 (methylacrylamide-GGGG-GQGFSYPYKAVFSTQ, Qiangyao Company, Shanghai, China) dissolved in 1 ml PBS and mixed evenly. Photoinducer lithium phenyl-2,4,6-trimethyl-benzoyl phosphonate (LAP, 0.1 wt%, Engineering For Life, Suzhou, China) was added to the solution. By exposed to a light-curing unit (Engineering For Life, Suzhou, China) for 20 s, the GelMA-BFP1 hydrogel was formed. In addition, the photocrosslinkable BFP1 was produced by mixing BFP1 with methylacrylamide. In same way, the unmodified BFP1 and GelMA were uniformly mixed in the above proportion, and GelMA/BFP1 hydrogel was formed after 20 s of illumination.

### Material characterization

#### Assessing the physicochemical structure of composite hydrogels

GelMA, GelMA/BFP1 and GelMA-BFP1 were characterized by Fourier transform infrared (FTIR) spectroscopy (Nicolet iS10, Waltham, MA, USA). The hydrogel samples were frozen overnight at −80°C, lyophilized, crushed and combined with potassium bromide. Each mixture was used to prepare discs using a tablet press. Crushed samples (400–4000 cm^−1^) were used with a resolution of 4 cm^−1^. Each FTIR measurement involved 32 scans of the sample, and the average of all scans yielded the reported spectra.

The hydrogels were freeze-dried for 24 h to obtain the test samples. Subsequently, the samples were sputter-coated with gold and imaged using a scanning electron microscope (SEM, Zeiss supra55, Oberkochen, Germany) to observe the surface microstructure of the material. The SEM images were analyzed using ImageJ software to determine the porosity of the freeze-dried hydrogels.

Three groups of hydrogels (1 ml, *n *=* *3) were immersed in PBS for 24 h and freeze-dried for 24 h to obtain dry samples. The swelling ratio was determined using the following formula: swelling ratio = (*W*_w_ − *W*_d_)/*W*_w_, where *W*_w_ and *W*_d_ are the wet and dry hydrogel weights, respectively.

To assess the degradation rates of the GelMA, GelMA/BFP1 and GelMA-BFP1 hydrogels, they were weighed (*W*_0_) and then submerged in PBS for 7, 14, 21, 28, 35 or 42 days. After each interval, the hydrogel samples were retrieved from the PBS, dried and weighed at specific degradation stages. The degradation ratio (DR) was calculated as DR = (*W*_0_ − *W*_t_)/*W*_0_ × 100%.

For mechanical testing, hydrogel samples with cylindrical form (10 mm diameter × 4.5 mm thick, *n *=* *5/group) were subjected to an unconfined compression examination with a strain rate of 1 mm/min (Instron 3400, MA, USA) after immersing them in 1 ml PBS for 24 h.

#### Bone-forming peptide-1 release behaviors of the hydrogels with gelatin methacrylate and bone-forming peptide-1

After labeling BFP1 and MA-BFP1 with FITC, 50 μg of fluorescently labeled polypeptides was mixed with 1 ml GelMA hydrogel and illuminated at 405 nm. Peptides were acquired from China Peptides Co. Ltd (Shanghai, China; purities > 98%). The hydrogels were submerged in 5 ml PBS at 4°C in the dark. Each day, 300 μl of PBS containing released, fluorescently tagged polypeptides was collected, after which 300 μl of PBS was added back. The fluorescence intensity (488/525 nm) of the collected samples was quantified using an EnSpire Multimode Plate Reader (PerkinElmer, Waltham, MA, USA), and polypeptide release was quantified using a standard curve for fluorescently labeled polypeptides.

Peptide retention in the hydrogels was quantified and visualized by preparing GelMA/BFP1 hydrogels with incorporated FITC-labeled BFP1 and GelMA-BFP1 hydrogels with included FITC-labeled MAA-BFP1, respectively. Each 200 μl hydrogel was cured on specialized scaffolds, with seven hydrogels analyzed per group. Subsequently, each hydrogel was placed in 2 ml PBS and gently agitated in a light-protected environment (at 37°C, 50 rpm). The hydrogels were later extracted and imaged under a fluorescence microscope (Leica, Wetzlar, Germany).

### Human dental pulp stem cell isolation

HDPSCs were isolated from healthy individuals after third-molar extraction with institutional review board permission (approval number K2022060). The procedure for the collection of isolated teeth complies with the ethical standards of the Declaration of Helsinki (2008) of the World Medical Association and has obtained the patient’s written consent. The hDPSCs were cultured in α-MEM with 10% fetal bovine serum (Gibco, Thermo Fisher Scientific, Inc., Waltham, MA, USA), 10 mg/ml streptomycin and 10 U/ml penicillin (Merck, Darmstadt, Germany). HDPSCs of passages 3–5 were used in this experiment.

### *In vitro* investigations

#### Cytocompatibility, cell viability and proliferation

To assess cytotoxicity, hDPSCs were three-dimensionally (3D) inoculated into the hydrogels at a density of 1.5 × 10^6^ cells/ml. The control group was treated with pure GelMA, and the polypeptide concentration in the GelMA/BFP1 and GelMA-BFP1 groups was 50 μg/ml. Cell viability was evaluated using a Live/Dead staining kit (Beyotime, Shanghai, China) and visualized under a fluorescence microscope.

Cell proliferation was assessed using cell counting kit-8 (CCK-8, Beyotime, Shanghai, China). Briefly, cells were inoculated into different hydrogels (1.5 × 10^6^ cells/ml). The samples were washed with PBS after 1, 3, 5 and 7 days of cultivation. Subsequently, 10 μl of CCK-8 solution was added to the samples, and they were incubated in the dark for 2 h. After transferring 100 μl of sample supernatant, the optical density (OD) values were measured at 450 nm using a microplate reader (BioTek Epoch, Vermont, USA).

#### Morphological and cytoskeletal observations

The morphology and cytoskeletons of hDPSCs cultured in scaffolds were examined using field-emission SEM and confocal laser-scanning microscopy (CLSM, Zeiss, LSM510, Oberkochen, Germany). The cells were immobilized for 2 h using a 2.5% (w/v) glutaraldehyde solution before the SEM examinations. The cells were dehydrated in increasing percentages of ethanol for 30 min. For CLSM, the cells were immobilized in 4% (w/v) paraformaldehyde for 30 min. Subsequently, they were permeabilized using a 0.1% (v/v) Triton X-100 (Merck, Darmstadt, Germany) for 10 min. To facilitate staining, the complexes were treated with phalloidin-Alexa Fluor 594 (Beyotime, Shanghai, China; 5 μg/ml). Following a PBS wash, the complexes were incubated with 4′,6-diamidino-2-phenylindole (DAPI, Merck, Darmstadt, Germany) at 10 μg/ml for 5 min. Finally, the stained samples were examined for intracellular signals via CLSM.

#### Quantitative real-time polymerase chain reaction analysis

To analyze the RNA-expression levels of osteogenic factors, hDPSCs were seeded in different hydrogels (1.5 × 10^6^ cells/ml) and cultured for 7 or 14 days. The hydrogels were incubated in osteogenic medium containing 100 nM dexamethasone, 50 μg/ml ascorbic acid and 10 mM β-glycerol phosphate. Subsequently, the medium was removed and the hydrogels were cracked with hydrogel lysate (Engineering For Life, Suzhou, China), centrifuged and washed to obtain the cells. Quantitative real-time polymerase chain reaction (qRT-PCR) analysis was conducted using SYBR Green (Takara, Japan) on an ABI 7500 RT-PCR instrument (Applied Biosystems, Waltham, USA). The comparative CT (2^ΔΔCT^) approach was used to assess differences in gene expression levels across groups.

#### Alkaline phosphatase and alizarin red S staining

The osteogenic-differentiation capabilities of the hydrogels were assessed via alkaline phosphatase (ALP) staining. Different hydrogels loaded with hDPSCs were seeded in osteogenic medium for 7 days. Next, the cells were fixed using 4% paraformaldehyde and stained using an ALP staining kit (Beyotime, Shanghai, China). Finally, the ALP-stained cultures were imaged using a fluorescence microscope.

To further verify the long-term bone-promoting effects of the composite hydrogel materials, different hydrogels loaded with hDPSCs were seeded in osteogenic medium for 21 days and stained with alizarin red S (Merck, Darmstadt, Germany). Cells were imaged using a fluorescence microscope. Alizarin red S-stained cultures were then immersed in 100 mM hexadecylpyridinium chloride (Merck, Darmstadt, Germany) for 1 h to solubilize the calcium-bound alizarin red S, and the absorbance was measured at 562 nm. Extracellular matrix mineralization was quantified for each sample.

### *In vivo* investigations

#### Surgical procedures

All animal experimental procedures were approved by the medical and experimental animal ethical review committee of China Medical University (No. CMU2023824) and were conducted in strict adherence to the Committee’s guidelines. To assess the bone-repairing capability of a cell-laden hydrogel with cross-linked polypeptides, 30 male Sprague Dawley rats (300 ± 28 g) were randomly assigned to five groups (i.e. the Blank, GelMA, GelMA/hDPSCs, GelMA/BFP1 and GelMA-BFP1 groups, *n *=* *6/group). A 5 mm annular defect was formed in the central area of each skull using a bonding drill after making a skin incision. The gel was injected into the bone defect, with the group not receiving a gel injection serving as the blank control. Subsequently, the area was irradiated at 405 nm for 20 s to solidify the gel, after which the skin was sutured. After surgery, the rats were placed in a constant temperature environment of 25°C and provided food, drinking water and timely bedding replacement.

#### New bone formation evaluated by micro-computed tomography

The rats were euthanized 4 and 8 weeks postoperatively, and their skulls were collected. The skulls were scanned and reconstructed via micro-CT (SkyScan 1276, Karlsruhe, Germany). CTvox software was utilized to determine the bone-reparation parameters, such as the new bone volume, bone volume/total volume (BV/TV) ratio and trabecular number (Tb.N), using a 5 mm cylindrical region of interest (ROI), which are presented as the mean ± SD of three rats.

#### Histological staining

The skulls were decalcified using a 10% ethylenediaminetetraacetic acid solution for 1 month after micro-CT scanning. Then the skulls were embedded in paraffin, sectioned into 5 μm slices and stained using hematoxylin and eosin (H&E) and Masson’s trichrome staining kit (Solarbio Life Sciences, Beijing, China). Rat heart, liver and kidney tissues were simultaneously obtained for H&E staining to evaluate the *in vivo* biocompatibility of the hydrogels.

#### Immunofluorescent staining

The tissue sections were rinsed with PBS and blocked with goat serum. Subsequently, the sections were incubated overnight at 4°C with primary antibodies against type-I rabbit collagen (Col-I) and osteocalcin (OCN), both of which are indicators of osteogenesis. The slices were then incubated with the appropriate secondary antibodies for 60 min (1:100, Beyotime, Shanghai, China). Finally, the slices were stained with DAPI, and images were taken using a fluorescence microscope. The acquired images were then subjected to quantitative analysis using ImageJ software.

### Statistical analysis

The *in vitro* assays were conducted in triplicate. The experimental data was analyzed using one-way analysis of variance and expressed as the mean ± SD. The significance levels are denoted as follows: *P <* *0.05, **P <* *0.01 and ***P <* *0.001.

## Results

### Characterization of GelMA, GelMA/BFP1 and GelMA-BFP1 hydrogels

**Figure 2.**
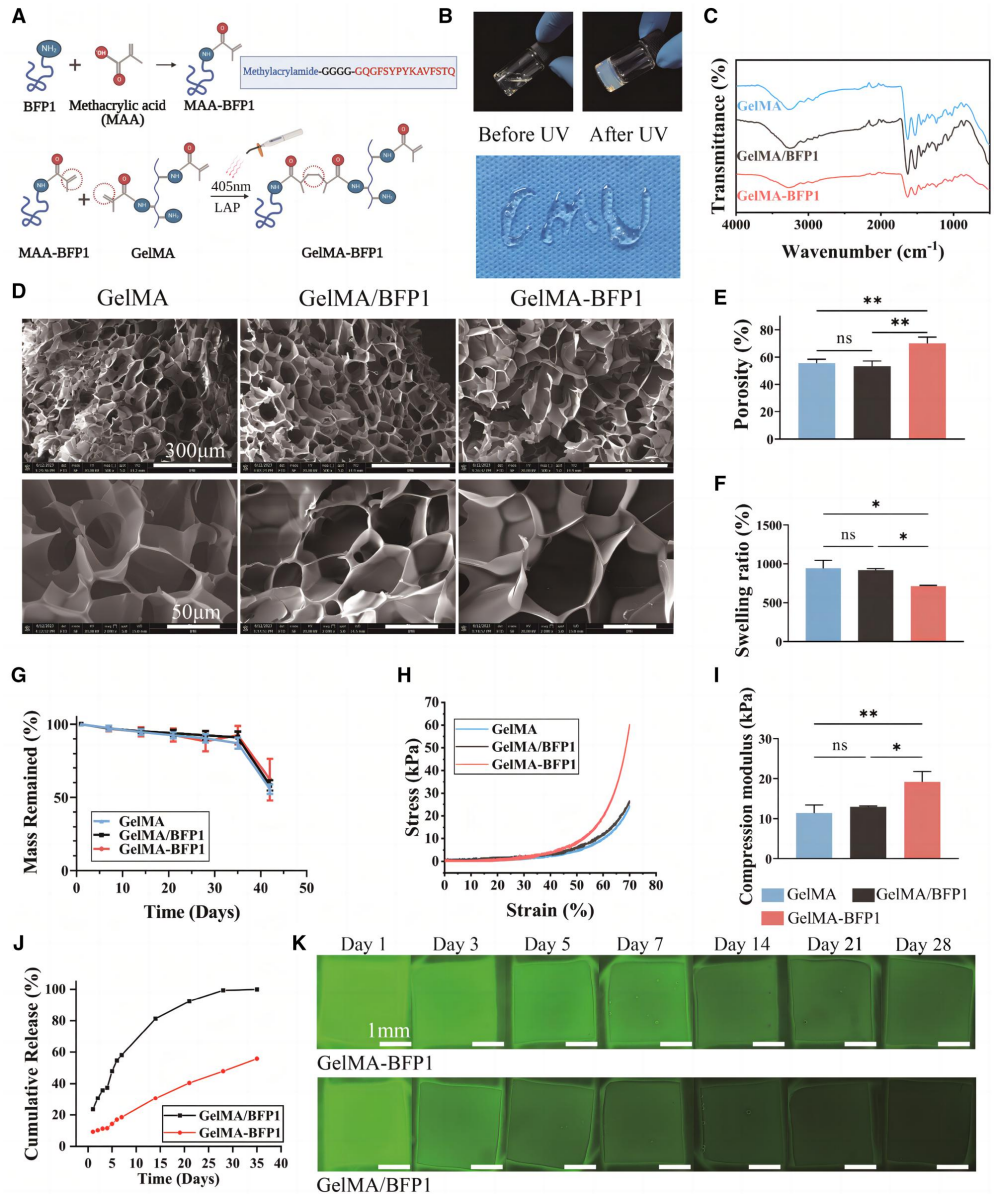
Characterization of different hydrogels. (**A**) The process used to prepare GelMA-BFP1 composite hydrogel. (**B**) Photographs of GelMA-BFP1 solution, the GelMA-BFP1 hydrogel formed after ultraviolet (UV) light exposure, and a fixed shape after photocrosslinking the GelMA-BFP1 hydrogel. (**C**) FTIR spectra, (**D**) SEM images, (**E**) porosity, (**F**) swelling, (**G**) degradation compressive stress–strain curve, (**H**) compressive stress–strain curve and (**I**) compression modulus of the GelMA, GelMA/BFP1 and GelMA-BFP1 hydrogels. (**J**) Cumulative release of BFP1 from the GelMA/BFP1 and GelMA-BFP1 hydrogels. (**K**) The fluorescence intensity of GelMA-BFP1 and GelMA/BFP1 hydrogels varies with time (*P <* *0.05, **P <* *0.01, ***P <* *0.001, *n *=* *3).

GelMA-BFP1 hydrogel was synthesized by dissolving methylacrylamide-BFP1, GelMA and a photoinitiator in PBS to prepare a hybrid solution of GelMA and MAA-BFP1. The hydrogel was formed after exposing the solution to 405 nm light for 20 s. Within the GelMA-BFP1 hydrogels, the BFP1 grafting reaction is chemically identical to the crosslinking reaction of methylated gelatin (Figure 2A). The resulting GelMA hydrogel and MAA-BFP1 mixture was highly fluid and injectable (Figure 2B). Unmodified BFP1 was added to the GelMA solution at the same concentration (50 μg/ml) to create the control GelMA/BFP1 hydrogel. Notably, the FTIR spectrum of GelMA-BFP1 was similar to those of GelMA/BFP1 and GelMA (Figure 2C). These observations indicated that the chemical bond formed by crosslinking BFP1 with GelMA was consistent with that formed by GelMA self-crosslinking.

Tissue-engineering scaffolds require large interconnected pores to facilitate cell growth and nutrient circulation [22, 23]. After freeze drying, structural analysis of the GelMA, GelMA/BFP1 and GelMA-BFP1 hydrogels revealed a porous interconnected structure characterized by irregularly shaped holes, predominantly elongated and spindle-shaped, with evident internal connectivity between the holes (Figure 2D). After assessing the SEM images with ImageJ software, the porosities of all three types of hydrogels were obtained. The quantified results highlighted significant variance in porosity between GelMA-BFP1 and GelMA/BFP1 (*P *<* *0.01), as well as among the GelMA hydrogels (Figure 2E).

The swelling ratios of the GelMA, GelMA/BFP1 and GelMA-BFP1 hydrogels were also assessed to determine their water-absorption capacities. Notably, GelMA-BFP1 demonstrated a slightly lower swelling ratio than GelMA and GelMA/BFP1 (Figure 2F), indicating that the crosslinking of GelMA-BFP1 may have resulted in a more compact internal structure and greater mechanical strength. The degradation of the hydrogels was further investigated by immersing GelMA, GelMA/BFP1 and GelMA-BFP1 hydrogels in PBS and recording the residual weights at predetermined intervals. It was found that the degradation rates among them were similar (Figure 2G) [24]. The conventional compressive-strength curves of all three types of hydrogels are depicted in Figure 2H. As expected, the GelMA-BFP1 hydrogels exhibited superior compression moduli and mechanical properties than those of the GelMA and GelMA/BFP1 groups (Figure 2I). Compared with the pure GelMA and GelMA/BFP1 hydrogels, the covalent cross-linkage of the BFP1 peptide substantially enhanced the compressive strength of the composite hydrogels. Notably, GelMA-BFP1 demonstrated a higher compression modulus (20 ± 2.2 kPa) than GelMA (12 ± 1.9 kPa) and GelMA/BFP1 (13 ± 0.2 kPa).

### Bone-forming peptide-1 release behaviors of the GelMA/BFP1 and GelMA-BFP1 hydrogels

The successful crosslinking of BFP1 into the hydrogel was further verified by calculating the BFP1-release rate, which was determined by measuring the fluorescence intensity of the supernatant over time [25]. BFP1 release was notably slower from GelMA-BFP1 than from GelMA/BFP1. By day 28, 80% of the peptides in the GelMA/BFP1 group were released. In contrast, less than 50% was released in the GelMA-BFP1 group after optical cross-linking (Figure 2J).

To investigate the effect of photocrosslinking on peptide release, peptide retention in hydrogels was photographed at different time intervals using a fluorescence microscope. The fluorescently labeled peptides were uniformly distributed within the hydrogels, and over time, the GelMA/BFP1 hydrogels showed a more rapid reduction in fluorescence intensity (Figure 2K). Thus, the polypeptides in GelMA-BFP1 exhibited prolonged retention within the hydrogel.

### Cytocompatibility of hydrogels with different bone-forming peptide-1 loading modes

**Figure 3.**
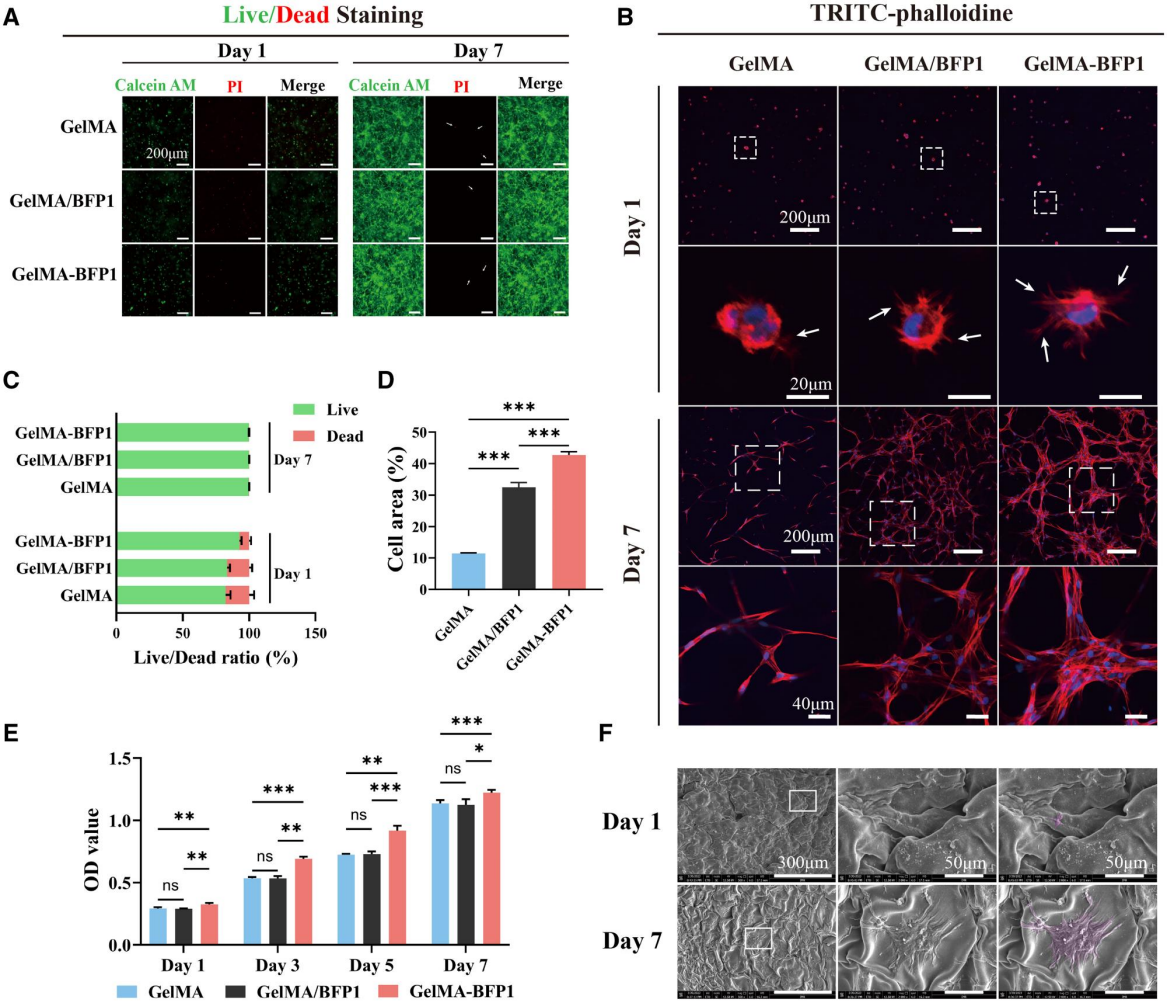
*In situ* morphology of hDPSCs encapsulated in different 3D matrixes. (**A**) Fluorescent images of live/dead staining. (**B**) Confocal-microscope images of cellular morphologies. Cell membrane and nuclei were stained with phalloidin-Alexa fluor 594 and DAPI. (**C**) Live/dead ratios are calculated from fluorescent images. (**D**) Cell-spreading areas of hDPSCs. (**E**) CCK-8 results of hDPSCs cultured for 1, 3, 5 and 7 days. (**F**) SEM images of hDPSCs encapsulated in GelMA-BFP1 hydrogels. (*P <* *0.05, **P <* *0.01, ***P <* *0.001, *n *=* *3).

Cell growth state and proliferation rates are key parameters for evaluating the biological properties of scaffolds [26]. In this study, hDPSCs were cultured in three different hydrogels, namely GelMA, GelMA/BFP1 and GelMA-BFP1 hydrogels. After 24 h, there were no disparities in the numbers of dead cells (red) across the three groups, and most cells were viable (green) (Figure 3A and C). The impact of peptide incorporation on cell growth was further assessed by quantitatively determining OD values through the performance of CCK-8 assays. The OD value increased with extended cell-culture periods, and the cell-proliferation rate was higher in the GelMA-BFP1 hydrogels. Specifically, the OD values of the GelMA-BFP1 hydrogels were higher than those of the GelMA and GelMA/BFP1 hydrogels after 1, 3, 5 and 7 days of culture. In contrast, the OD values of GelMA and GelMA/BFP1 hydrogels were not significantly different at those time points (Figure 3E). These findings suggest that the cross-linked scaffold was not cytotoxic and that it promoted cell proliferation. These findings further suggest that the GelMA-BFP1 hydrogel exhibited a superior ability to enhance cell proliferation during the period studied.

### Cellular morphology and cytoskeletal observations

The growth of cells in the 3D culture system we developed was validated by conducting phalloidin-Alexa Fluor 594 staining. From the CLSM micrographs of the phalloidin-Alexa Fluor 594 stained samples, it is evident that hDPSCs encapsulated within the GelMA-BFP1 hydrogel exhibited significantly increased diffusion. Notably, on day 1, hDPSCs cultured in the GelMA-BFP1 hydrogel displayed a well-distributed morphology characterized by distinct F-actin stress fibers. In contrast, hDPSCs encapsulated in the GelMA hydrogel exhibited a round cell morphology without significant stress fibers. Following a 5-day growth period, cells in the GelMA hydrogel exhibited limited diffusion and acquired a filamentous form. In contrast, the GelMA-BFP1 hydrogel demonstrated thicker network fibers, resulting from hDPSCs cross-linking through the cell pseudopods. In contrast, the GelMA hydrogel did not exhibit the same level of cross-linking or facilitate the formation of thicker network fibers (Figure 3B). These findings highlight the advantage of incorporating BFP1 into GelMA hydrogels, as it enables the development of a more favorable environment for hDPSCs, enhancing their overall performance and potential for various applications. This network structure facilitated enhanced biomechanical and biochemical signals from the surrounding microenvironment, contributing to improved viability, diffusion and hDPSCs proliferation [27]. Additionally, this morphology may capture more peptides from the matrix through additional cytopeptide cross-linking networks [28]. Moreover, the analysis of the CLSM 3D-projection images shown in Figure 3D indicated that after 5 days of culture, GelMA-BFP1 demonstrated a higher cell density and larger cell area than the GelMA and GelMA/BFP1 hydrogels. Thus, the composite structure of the GelMA-BFP1 hydrogel may facilitate cell growth and proliferation. The CLSM results confirmed that the composite structure of the GelMA-BFP1 hydrogel fostered hDPSCs adhesion, ultimately resulting in improved cell proliferation.

SEM images were obtained to visualize the growth of cells within the GelMA-BFP1 hydrogel. On day 1 in 3D culture, the cells were individually distributed within the hydrogel and exhibited conspicuous pseudopods. By day 7, the hDPSCs demonstrated robust proliferation. Furthermore, they exhibited an expanded morphology with more pseudopods (Figure 3F). Thus, the GelMA-BFP1 hydrogel potentially supported cell growth and proliferation. Pseudopods exhibited cell migration and exploration of the surrounding microenvironments [29]. The substantial increase in cell number, expanded morphology and pseudopods further supported the notion of successful cell proliferation and the potential for extensive cellular interactions within the GelMA-BFP1 hydrogel. The SEM images provided valuable insights into cellular behavior and growth dynamics within the GelMA-BFP1 hydrogel, highlighting its potential as a scaffold for bone tissue engineering.

### Osteogenic effects of composite hydrogels *in vitro*

**Figure 4.**
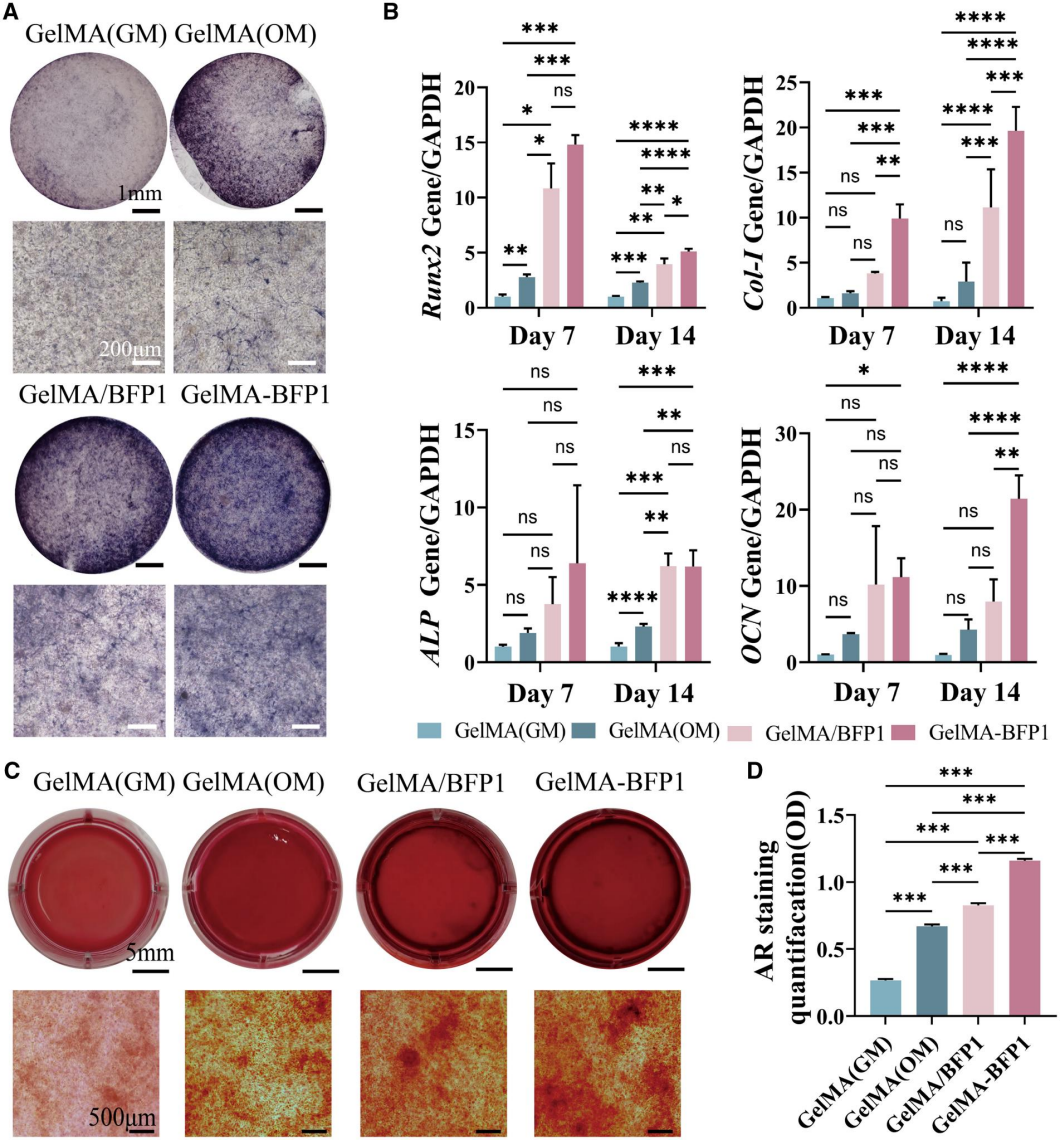
The osteogenic properties of hydrogels. (**A**) Representative ALP-staining images after 7 days. (**B**) The relative gene expression levels of *Runx2*, *Col-I*, *ALP* and *OCN* in hDPSCs were analyzed by qRT-PCR after 7 and 14 days of osteogenic induction (GM: growth medium; OM: osteogenic medium). (**C**) Representative images of alizarin red S staining after 21 days of osteogenic induction. (**D**) Quantitative analysis results of alizarin red S staining (*P <* *0.05, **P <* *0.01, ***P <* *0.001, *n *=* *5).

To examine the bone-promoting effects of the GelMA-BFP1 hydrogels, hDPSCs were cultured in GelMA/BFP1 and GelMA-BFP1 hydrogels. A GelMA hydrogel without the BFP1 polypeptide was used as a control. ALP expression was assessed 7 days after osteogenic induction. The results revealed more ALP-positive cells in the GelMA-BFP1 group, with a darker staining color than in the other two groups (Figure 4A). These findings indicated that the GelMA-BFP1 hydrogel exhibited better bone inductivity.

The impact of GelMA-BFP1 on bone formation was investigated by assessing the gene-expression levels of osteogenic markers in hDPSCs using qRT-PCR at 7 and 14 days. The osteogenic genes studied included runt-related transcription factor 2 (*Runx2*), *Col-I*, *ALP* and *OCN*. The results indicated that both the GelMA/BFP1 and GelMA-BFP1 groups had significantly higher *Runx2*, *Col-I* and *ALP* levels than the GelMA group at both time points (*P *<* *0.05, Figure 4B).

The GelMA-BFP1 group expressed significantly higher levels (*P *<* *0.05) of *Runx2*, *Col-I* and *ALP* than the GelMA/BFP1 group, indicating that the bone-promoting effect of BFP1 on hDPSCs was further enhanced after crosslinking with the polypeptide hydrogel. On day 14, the GelMA-BFP1 group had significantly higher *OCN*-expression levels than the GelMA/BFP1 group (*P *<* *0.05), indicating that the crosslinking hydrogel of the polypeptide prolonged the osteogenic effect of the polypeptide and enhanced its overall efficacy. The gene-expression patterns of bone-related proteins further underscore the efficiency and capacity of GelMA-BFP1 hydrogels in accelerating the osteogenic differentiation of hDPSCs. Furthermore, BFP1 crosslinking inside the GelMA hydrogel complex increased the bone-promoting effect of hDPSCs versus BFP1 treatment alone. Thus, the scaffolds potentially enhance osteogenic differentiation.

Alizarin Red S staining was performed to assess the development of calcified clusters within each experimental group. After 21 days of osteogenic induction, the four groups of hydrogel complexes were stained with alizarin red S. The property of the hydrogel itself to adsorb the dye caused all four groups of hydrogels to show a broad red color, while a large number of reddish-brown calcium nodules could be observed under the microscope in the GelMA-BFP1 group (Figure 4C). Additionally, quantitative analysis of alizarin red S staining further corroborated the superior osteogenic effect of the GelMA-BFP1 group related to that of the other two groups (*P *<* *0.05, Figure 4D). This finding provides additional evidence supporting the robust and durable osteogenic ability of GelMA-BFP1.

### *In vivo* osteogenesis of human dental pulp stem cells encapsulated in composite hydrogels

**Figure 5.**
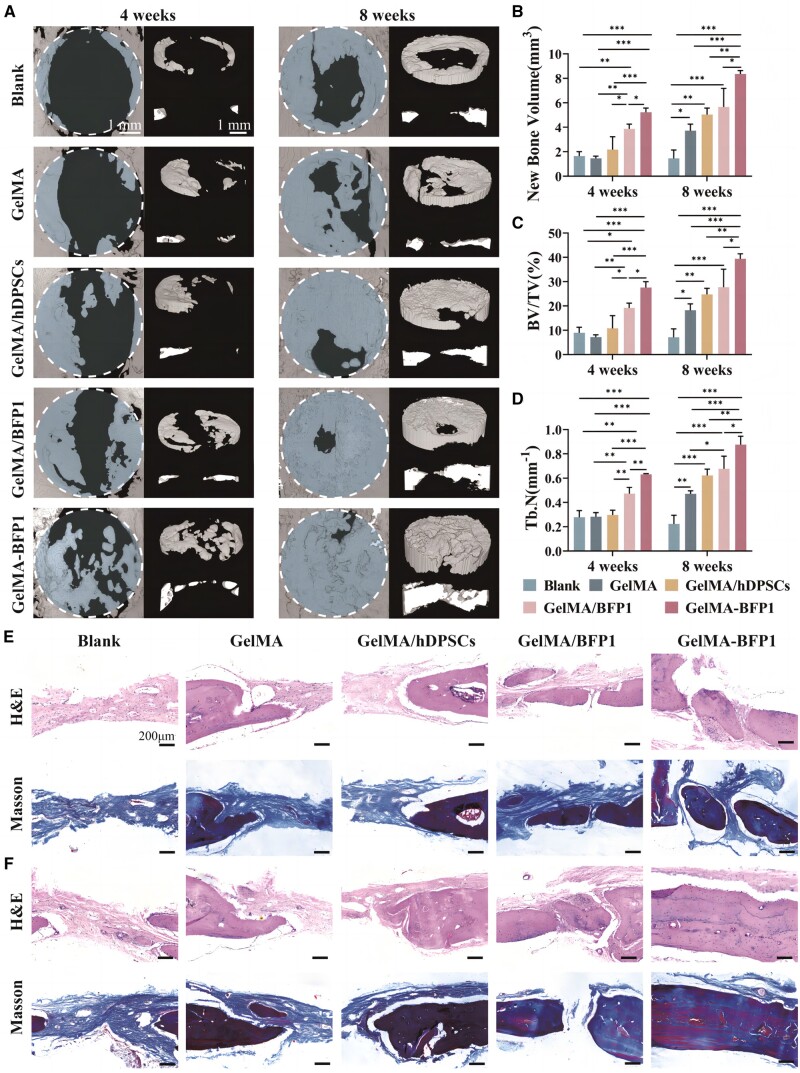
Bone formation was evaluated at 4 and 8 weeks after implantation with GelMA, GelMA/hDPSCs, GelMA/BFP1 hydrogels or GelMA-BFP1 hydrogels, or a blank control group. (**A**) Three-dimensional reconstruction of rat skulls. (**B**–**D**) Quantitative analysis of the new BV/TV ratios in the defects. (**E**, **F**) H&E staining and Masson’s trichrome staining demonstrated the relative amounts of new bone formation at 4 and 8 weeks post-surgery in the blank group and the other four experimental groups (*P <* *0.05, **P <* *0.01, ***P <* *0.001, *n *=* *3).

Considering the *in vitro* data, 5-mm defect models were established with rat skulls to investigate the osteogenic capability of GelMA-BFP1 scaffolds *in vivo*. Micro-CT analysis revealed limited marginal bone formation in the blank and GelMA-treated groups. The other groups showed substantial new bone formation after 4 weeks postoperatively. In the GelMA-BFP1 group, sporadic bone filling occurred at the center of the bone defect. In contrast, discontinuous bone formation occurred, potentially owing to the enhanced osteogenic effect of hDPSCs in the scaffold under continuous BFP1 induction. Subsequently, the cell mass gradually formed independent bone islands, which fused to repair the bone defects. At 8 weeks, both the GelMA and GelMA/hDPSCs groups demonstrated marginal bone fusion, indicating that new bone production increased. The GelMA/BFP1 and GelMA-BFP1 groups exhibited better repair effects than the GelMA group, with the GelMA-BFP1 group showing thicker new bones. Surprisingly, island osteogenesis occurred in the GelMA-BFP1 group, in which hDPSCs proliferated to form independent osteogenic units and eventually bone islands (Figure 5A).

Our quantitative examination of the new bone volumes confirmed that the GelMA-BFP1 group showed significantly more new bone formation (*P *<* *0.05) at both 4 and 8 weeks post-surgery (Figure 5B). The BV/TV ratio indicated that the GelMA-BFP1 group had significantly higher bone percentages (*P *<* *0.05) at 4 and 8 weeks post-surgery (Figure 5C) versus the other groups. After 4 weeks, the GelMA-BFP1 group had a mean BV/TV ratio of 28 ± 3%, which was 3.1 times higher than that of the blank group. After an 8-week period, the GelMA-BFP1 scaffold exhibited the highest BV/TV ratio (39 ± 2%). Thus, the bone-regeneration efficacy of the GelMA-BFP1 group was enhanced, resulting in substantial production of mature bone tissues. Trabecular bone, an extension of cortical bone into cancellous bone, creates an uneven 3D network within the marrow cavity and facilitates hematopoiesis [30, 31]. Hence, assessing the Tb.N is crucial for evaluating bone regeneration. The GelMA-BFP1 scaffold group had the highest Tb.N value (Figure 5D), demonstrating the excellent *in situ* osteogenic properties of the GelMA-BFP1 hydrogel.

Inspired by the micro-CT scan images, H&E and Masson’s trichrome staining was performed after 4 and 8 weeks to obtain a more detailed understanding of the histomorphology of the bone defects in each group following the healing process. The defects in the blank group were primarily occupied by loose fibrous connective tissue after 4 weeks (Figure 5E). The GelMA, GelMA/hDPSCs and GelMA/BFP1 groups exhibited new bone development, although they lacked fully mature bone tissues. The regeneration of new bone tissue in the GelMA-BFP1 group showed the characteristics of bone island multi-site osteogenesis. Therefore, the GelMA-BFP1 hydrogel scaffold imbued with hDPSCs could potentially enhance *in situ* and multi-site bone regeneration within rat skull defects. As the post-operative duration reached 8 weeks, the GelMA, GelMA/hDPSCs and GelMA/BFP1 groups still had abundant fibrous tissues, whereas the bone defects in the GelMA-BFP1 group was practically filled with freshly regenerated bone tissue. The newly produced bone tissue formed numerous new connections with the original bone, forming a continuous and dense bone tissue (Figure 5F).

Masson’s trichrome staining, a specialized staining technique for collagen fibers, was also used to assess the bone regeneration capability of the GelMA-BFP1 scaffold. The characteristics of bone-island osteogenesis were observed in the GelMA-BFP1 group at 4 weeks, with a few red areas indicating that mature mineralized bone had formed during the regeneration process (Figure 5E). After 8 weeks, Masson’s trichrome staining revealed that only a minor amount of bone formation occurred with the blank group, whereas the GelMA-BFP1 group exhibited a red: blue transition, indicating that the GelMA-BFP1 scaffold stimulated mature and mineralized bone growth within the defect region (Figure 5F). Accordingly, the GelMA-BFP1 hydrogels loaded with hDPSCs exhibited strong bone inductivity.

**Figure 6.**
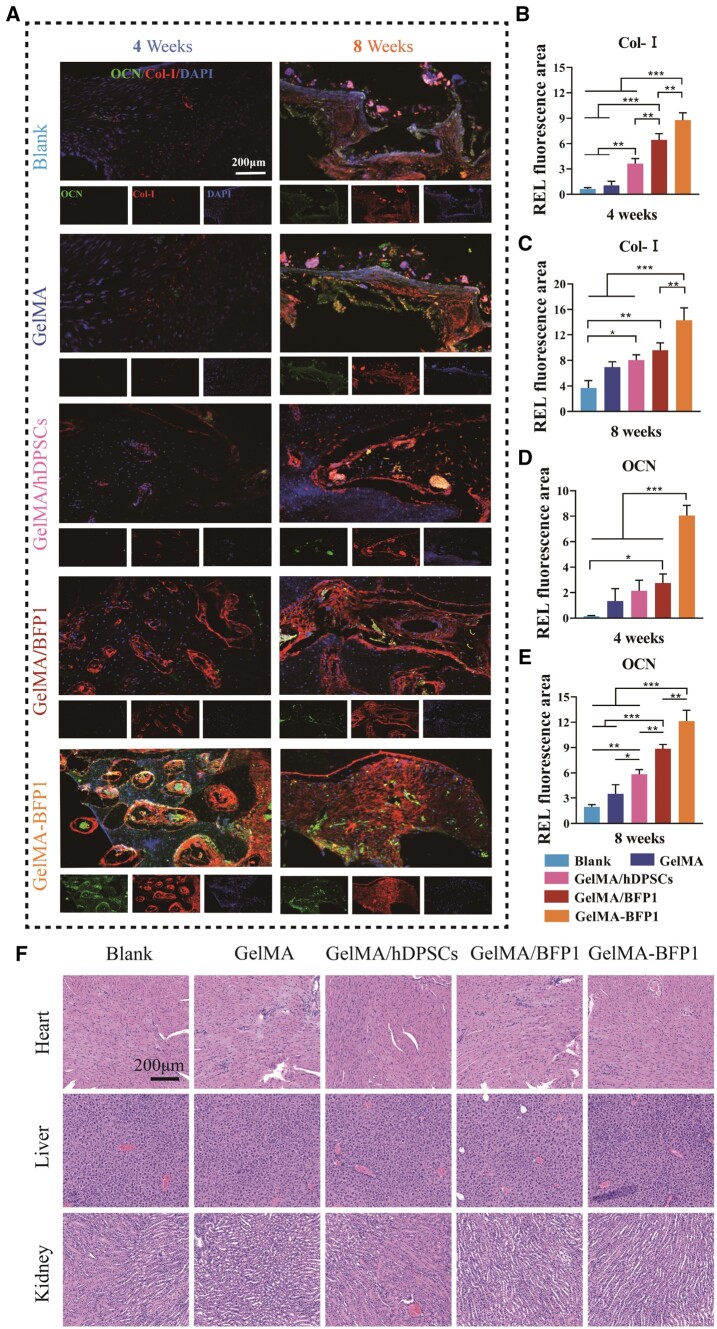
Histological evaluations of osteogenesis. (**A**) Representative immunofluorescence images of OCN, Col-I and cell nuclei stained at 4 and 8 weeks. (**B**–**E**) Quantitative analysis of all positively stained areas. (**F**) H&E staining of rat heart, liver and kidney sections after gel implantation in skull defects (*P <* *0.05, **P <* *0.01, ***P <* *0.001, *n *=* *3).

The biocompatibility of the biohybrid hydrogels was assessed *in vivo* by H&E staining of rat heart, liver and kidney tissues. These findings demonstrated the absence of any anomalies in these tissues, suggesting that these hydrogels exhibited no *in vivo* toxicity (Figure 6F).

Immunohistochemical staining revealed correlations between biomarker-expression levels in the GelMA-BFP1 scaffold and bone regeneration *in vivo*. OCN and Col-I immunofluorescence staining revealed that the GelMA-BFP1 group exhibited the most positive results (Figure 6A). Col-I is a vital marker for extracellular matrix production and the mechanical strength of bones [32]. The more the collagen fibers are organized, the stronger the newly formed bone becomes [33]. Consequently, Col-I levels were measured to determine the osteogenic potential of the biohybrid hydrogels. The GelMA-BFP1 group showed the greatest Col-I staining (red) and exhibited notable disparity versus the other groups (*P *<* *0.01; Figure 6B and C). OCN is the most abundant non-collagenous bone matrix protein and is specifically produced by osteoblasts [34]. Notably, green fluorescence (OCN staining) was distributed in flaky clumps at 4 weeks, with the highest intensity located at the clump centers, suggesting that OCN was highly expressed in the GelMA-BFP1 group. After 8 weeks, the GelMA-BFP1 group showed high green fluorescence, demonstrating the formation of lamellar bone-like structures. The blank, GelMA, GelMA/hDPSCs and GelMA/BFP1 groups exhibited significantly lower cord-like green fluorescence (Figure 6A). The results of our 4-week quantitative analysis revealed that OCN expression in the cells embedded within the hydrogel of the GelMA-BFP1 group was far greater than that in the other four groups (*P *<* *0.001; Figure 6D). Quantitative analysis revealed that the ratio between the GelMA-BFP1 and blank groups was approximately 6:1 at 8 weeks post-surgery (Figure 6E).

## Discussion

Stem cell-based biomaterials are currently employed in bone tissue engineering as a cutting-edge strategy for treating and repairing maxillofacial bone defects [35–37]. Zuba-Surma *et al.* [38] evaluated the osteogenic differentiation of 3D-cultured hDPSCs. Their findings suggested that under simulated hypoxic conditions, hDPSCs proliferation and metabolic activity decreased *in vitro*. In contrast, increased osteogenic differentiation occurred. These findings underscore the need to focus on methods used to construct scaffold materials, which is crucial when hDPSCs loaded into engineered bone tissues are used to repair maxillofacial bone defects [39]. Previously, amplifying stem cells and inducing their differentiation were predominantly accomplished by pre-differentiating stem cells *in vitro* [40, 41]. A significant challenge in stem cell-based bone tissue engineering is ensuring that the biomaterial scaffolding selectively promotes early stem cell proliferation *in vivo*. This process must be followed by promoting the osteogenic differentiation of these stem cells, with the ultimately goal of achieving *in situ* osteogenesis [42]. However, attaining effective bone formation remains a bottleneck. In this study, a biomaterial system was designed to enhance early stem cell proliferation. This system effectively harnessed the power of a minimal number of stem cells to produce robust and enduring effects on bone repair. Our findings pave the way for an efficient bone-repair method that reduces the need for an extensive volume of stem cells, making it a potentially pivotal advancement in the field of stomatology.

Stem cell-based bone engineering is a viable, rapid and effective strategy for bone regeneration, whereas the process requires large numbers of stem cells [10, 43]. HDPSCs are advantageous over bone marrow-derived mesenchymal stem cells (BMMSCs) because they are simpler to sample, easier to amplify and preserve and exhibit higher vitality [4]. Moreover, some scholars have confirmed that hDPSCs has strong osteogenic activity by loading hDPSCs into different hydrogel scaffolds for osteogenic induction *in vitro* [44, 45]. However, the limited quantity of dental pulp tissue that can be obtained after extraction poses a significant hurdle in harnessing hDPSCs for clinical applications. Therefore, optimizing the biomaterial design to maximize bone-repair efficiency with minimum stem cell loading is pivotal for increasing the feasibility of stem cell-based bone-regeneration treatments. BFP1 is a truncated peptide derived from immature BMP-7 that possesses more potent osteogenic activity than its mature full-length counterpart [13]. The results of numerous studies aimed at inducing osteogenesis *in vitro* have shown that adding low doses of BFP1 to biomimetic scaffolds can potentially promote osteogenesis [46, 47]. Inspired by the work of West *et al.* [48] and Higuchi *et al.* [49], we designed a methacrylated BFP1 molecule (MAA-BFP1) that showed comparable bioactivity relative to the unmodified counterpart. Under the action of photoinitiator, the covalent binding of MAA-BFP1 to the GelMA hydrogel with carbon-carbon double bonds occurred after 20 s of irradiation with 405 nm blue light, which resulted in the anchoring of BFP1 within the hydrogel. BFP1 was then released in minute amounts, and its low concentration effectively stimulated the rapid proliferation of seed cells. Upon entering the differentiation phase, BFP1 interacted quickly and sustainably with receptors on the membrane surfaces of hDPSCs, thereby promoting osteogenic differentiation. This hydrogel complex was named the GelMA-BFP1 group. In contrast, the unmodified BFP1 peptide was added to the GelMA hydrogel in the form of physical mixing, and the peptide was mixed well with the hydrogel and then light exposed for 20 s. The hydrogel complex formed was named the GelMA/BFP1 group.

Cell proliferation and adhesion are critical indicators for evaluating the biocompatibility of biomaterials, and the pore size of a material is important for cell survival [50, 51]. Previous findings showed that a pore size of 100–300 μm facilitated cell proliferation and substance exchange [52]. Owing to its excellent biocompatibility, GelMA hydrogels have been extensively employed in 3D culture and for bone induction with mesenchymal stem cells (MSCs) [53]. However, conventional GelMA hydrogels typically possess small pore sizes, which often restrict the survival and proliferation of the encapsulated cells. This restriction compromises the efficacy of osteogenic differentiation, necessitating an increased quantity of stem cells. Additionally, cells inside hydrogels may undergo apoptosis owing to hypoxia, which poses a severe challenge to stem cell-based tissue engineering. Our results demonstrated that the pore size of the GelMA-BFP1 hydrogel was approximately twice as large as that of pure GelMA. The larger pore size facilitated the exchange of oxygen and nutrients, which significantly enhanced hDPSCs proliferation and adhesion. The results of the live/dead and CCK-8 assays indicated that the GelMA-BFP1 hydrogel significantly affected cell proliferation during the proliferation phase. After rhodamine-phalloidin staining, our CLSM microimages revealed that the hDPSCs encapsulated in the GelMA-BFP1 hydrogel exhibited superior adhesion properties, higher cell densities and larger cell-spreading areas. In addition, our SEM results revealed increases in the cell size and number of pseudopodia formed. Collectively, these findings demonstrate that the composite structure of the GelMA-BFP1 hydrogel better supported cell growth, proliferation and adhesion, thus offering an advantageous environment for cell development and proliferation.

Bone regeneration involves the regulation of several osteogenic genes [54]. *ALP*, *OCN*, *Runx2* and *Col-I* have been described as master transcription factors for osteogenesis [55]. *ALP* can hydrolyze organic phosphorus, leading to an increased ${\text{PO}}_{4}^{3-}$ concentration, which facilitates Ca^2+^ deposition and subsequently regulates bone mineralization [56]. *OCN* levels correlate with the bone mass density and turnover [57], whereas *Runx2* and *Col-I* are essential for osteoblast differentiation and chondrocyte maturation [58, 59]. The expression of genes associated with osteogenic markers in hDPSCs over 7 and 14 days was assessed by performing qRT-PCR. The results demonstrated progressive increases in *Runx2*, *Col-I* and *ALP* expression at specific time points in the GelMA, GelMA/BFP1 and GelMA-BFP1 groups. Additionally, on day 14, the GelMA-BFP1 group exhibited significantly higher *OCN* expression than the GelMA/BFP1 group. This finding indicates a heightened degree of osteogenic differentiation within the GelMA-BFP1 hydrogel, suggesting that the chemically crosslinked hydrogel prolonged the osteogenic effects of the peptide, enhancing its overall bone-promoting efficacy. Consequently, a strategy was devised to enable a limited number of stem cells to attain enhanced osteogenic outcomes.

In light of the *in vitro* findings, *in vivo* experiments were carried out on adult rats. It was observed that the group treated with cell-loaded GelMA-BFP1 hydrogel exhibited the most pronounced positive impact on the repair of skull defects. By week 4, a significant enhancement in bone formation was detected in the GelMA-BFP1 group. This newly formed bone demonstrated a disjointed morphology, leading to multi-site bone regeneration. These findings underscore the occurrence of *in situ* bone regeneration in this group. In contrast, the control group displayed minimal new bone formation, with only a small amount of new bone produced around the bone defect. Traditional bone-tissue regeneration strategies often result in the formation of bone tissue through osteogenesis, converging from the perimeter towards the center [60]. These results suggest that under the guidance of the GelMA-BFP1 scaffold, hDPSCs successfully differentiated into osteoblasts after a period of proliferation, thereby achieving the anticipated *in situ* osteogenic effects. This unique osteogenic phenomenon, which differed from the osteogenic effects observed in previous research, may have been caused by the slow and sustained release of BFP1. This process likely enhanced hDPSCs proliferation and cellular adhesion, amplifying pseudopodia formation and thus reinforcing the overall bone-formation process. Furthermore, hDPSCs can secrete exosomes, promote osteogenic differentiation and MSC recruitment to bone defects and accelerate bone regeneration [61, 62]. Compared to earlier hydrogels, the GelMA-BFP1 hydrogel loaded with hDPSCs showed an impressive capacity to concurrently regenerate bone at multiple sites [63]. This innovative approach substantially augments both the velocity and quality of bone formation, thereby offering notable improvements in bone-regeneration research. Thus, we believe that the GelMA-BFP1 hydrogel loaded with hDPSCs constructed in this study can potentially make a critically vital contribution to clinical bone augmentation in the future.

## Conclusions

In conclusion, a GelMA-BFP1 photocrosslinking scaffold was constructed by chemically crosslinking BFP1 to achieve sustained release of the peptide and maximize the osteogenic capacity of hDPSCs. Cells were loaded into this scaffold in 3D at a density of 1.5 × 10^6^ cells/ml, and finally repaired rat cranial defects using the cell-loaded peptide-crosslinked hydrogel composite scaffold. Based on the osteogenic amplification effect of this system, a synergistic multi-site osteogenic effect was achieved, resulting in the formation of ‘bone island’-like osteogenesis. More importantly, critical bone defects in the rat cranium were efficiently regenerated using hDPSCs derived from a single tooth, which significantly improved the issue of insufficient donor volume in stem cell therapy.
